# Current status of xenotransplantation research and the strategies for preventing xenograft rejection

**DOI:** 10.3389/fimmu.2022.928173

**Published:** 2022-07-28

**Authors:** Qiao Zhou, Ting Li, Kaiwen Wang, Qi Zhang, Zhuowen Geng, Shaoping Deng, Chunming Cheng, Yi Wang

**Affiliations:** ^1^ Department of Rheumatology and Immunology, Sichuan Provincial People’s Hospital, University of Electronic Science and Technology of China, Chengdu, China; ^2^ Clinical Immunology Translational Medicine Key Laboratory of Sichuan Province, Sichuan Provincial People’s Hospital, University of Electronic Science and Technology of China, Chengdu, China; ^3^ Chinese Academy of Sciences Sichuan Translational Medicine Research Hospital, Chengdu, China; ^4^ Department of Rheumatology, Wenjiang District People’s Hospital, Chengdu, China; ^5^ School of Medicine, Faculty of Medicine and Health, The University of Leeds, Leeds, United Kingdom; ^6^ School of Medicine, University of Electronics and Technology of China, Chengdu, China; ^7^ Institute of Organ Transplantation, Sichuan Academy of Medical Science and Sichuan Provincial People’s Hospital, Chengdu, China; ^8^ Department of Radiation Oncology, James Comprehensive Cancer Center and College of Medicine at The Ohio State University, Columbus, OH, United States; ^9^ Department of Critical Care Medicine, Sichuan Academy of Medical Science and Sichuan Provincial People's Hospital, Chengdu, China

**Keywords:** Xenotransplantation, hyperacute rejection, delayed xenograft rejection, chronic rejection, glucocorticoids, immunosuppressants

## Abstract

Transplantation is often the last resort for end-stage organ failures, e.g., kidney, liver, heart, lung, and pancreas. The shortage of donor organs is the main limiting factor for successful transplantation in humans. Except living donations, other alternatives are needed, e.g., xenotransplantation of pig organs. However, immune rejection remains the major challenge to overcome in xenotransplantation. There are three different xenogeneic types of rejections, based on the responses and mechanisms involved. It includes hyperacute rejection (HAR), delayed xenograft rejection (DXR) and chronic rejection. DXR, sometimes involves acute humoral xenograft rejection (AHR) and cellular xenograft rejection (CXR), which cannot be strictly distinguished from each other in pathological process. In this review, we comprehensively discussed the mechanism of these immunological rejections and summarized the strategies for preventing them, such as generation of gene knock out donors by different genome editing tools and the use of immunosuppressive regimens. We also addressed organ-specific barriers and challenges needed to pave the way for clinical xenotransplantation. Taken together, this information will benefit the current immunological research in the field of xenotransplantation.

## 1 Introduction

Organ failures, which are usually a consequence of diseases, trauma, or alcohol/drug abuse, represent the top causes of mortality in most population groups (1). A recent population-based cohort study covering a 3-year period and involving 9,187 adult patients admitted at the emergency department of the Odense University Hospital, Denmark, indicated that the one-year all-cause mortality of organ failure was 29.8% (2). Another study, involving a cohort of 1,023 patients sequentially admitted at ten Scottish intensive care units, revealed a one-year overall mortality of 46.5% (3). Organ transplantation is the ideal treatment for most end-stage organ failure affecting the heart, lungs, kidneys, liver, and in certain cases, the pancreas. However, the demand of human organs for transplantation purpose exceeds by far the number of organ donations, which limits this procedure in general clinical practice. The World Health Organization (Geneva) estimates that only 10% of the worldwide need for organ transplantation is being met (4). Several countries around the world have implemented different strategies to overcome human organ shortage, including financial incentives in the United States and China (5, 6), or campaigns to increase public awareness (7). France and other European countries recently declared all citizen as donors by default in case of death or brain death unless they opted out during their lifetime. Other approaches are also explored to enable the use of animals’ organs and tissues, and are referred to as xenotransplantation. In addition to addressing organ shortage issues, xenotransplants could allow for tailored transplantation and meet specific patients’ needs, or offer more flexible schedules than that imposed by organ donations, usually performed on short notice (8).

The prominent pathobiological barriers to successful clinical use of xenotransplantation include the rapid activation of innate cellular responses against the graft, and at later stage, further rejection of the organ by the adaptive immune system (9, 10). As important, coagulation dysregulation and inflammation intervene in the rejection process. The long-term survival of the grafts depends on how to reduce or even avoid the occurrence of rejection, which in turn requires a deep understanding of these different rejection mechanisms. In this review, we illustrate in detail from current literatures these different mechanisms in rejection processes and the corresponding strategies to overcome them. We hope this comprehensive overview will bring new insights into current immunological research related to xenotransplantation and future directions towards its application.

## 2 Mechanism of xenograft rejection

### 2.1 Hyperacute rejection

**Figure 1:**
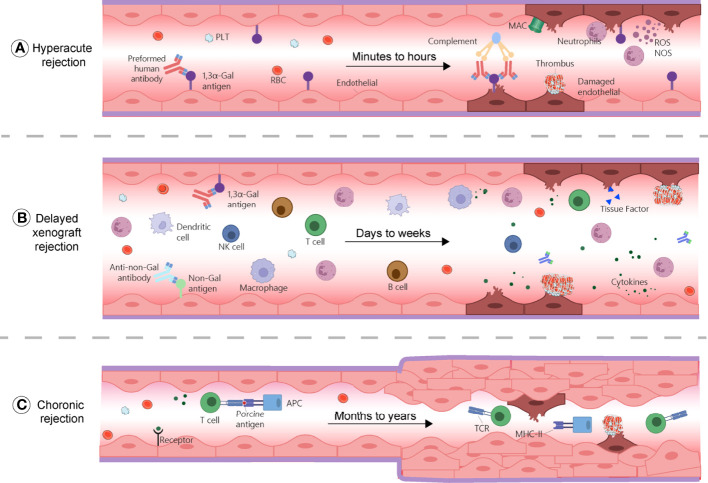
Mechanisms of rejections during xenotransplantation. **(A)** Hyperacute rejection occurs within minutes to hours and is caused by the binding of the host’s pre-existing antibodies to α-Gal antigens on the graft, which results in complement activation and membrane attacking complex (MAC) formation. This reaction causes endothelial cells lysis, fibrinoid occlusion, and vasculature destruction. Neutrophils, through the production of ROS and NOS also contribute to this process. **(B)** Delayed xenograft rejection (DXR) occurs within days to weeks and include acute humoral xenograft rejection (AHXR), cellular xenograft rejection, and coagulation dysregulation. AHXR is antibody-mediated and involve non-Gal antibodies and α-Gal antibodies reactivity against non-Gal epitopes and α-Gal of the graft. Various innate and adaptive immune cells, proinflammatory cytokines, and coagulation dysregulation contribute to rejection, resulting in massive deposition of immunoglobins, fibrin, endothelial cell lysis, and interstitial bleeding. **(C)** Chronic rejection occurs within months to years. Xenoantigens are surveyed by host APCs and presented to T cells, leading to their activation and triggering inflammatory cascades, characterized by thrombotic microangiopathy, proliferation of the graft vascular endothelial cells, vessel narrowing, and interstitial fibrosis. APC, antigen presenting cell; MAC, membrane attacking complex; MHC-II, major histocompatibility complex class II; NK cell, natural killer cell; NOS, nitric oxide species; PLT, platelet; RBC, red blood cells; ROS, reactive oxygen species; TCR, T cell receptor.

HAR generally defines as a graft destruction occurring within 24 hours and that usually lasts for few minutes to hours. It is caused by the binding of human or non-human primate (NHP) pre-existing antibodies against graft antigens (**Figure 1A**) (11). Among these antibodies, most frequent IgMs and IgGs recognize galactose-α 1,3-galactose (α-Gal) residues added on glycoproteins and glycolipids by the α1,3 galactosyltransferase (*α1,3GT*) present in the genomes of non-primates and New World monkeys (platyrrhine primates living in South and Central America, including howlers, spider monkeys, and woolly monkeys) (12, 13). Humans, Old World monkeys (catarrhine primates living in African, Asian and Europe, including baboons, colobuses and mandrills), and apes lack α-Gal epitopes because their *α1,3GT* gene is affected by a loss-of-function mutation (14). In addition, about 70–90% of the antibodies produced by these species target α-Gal epitopes specifically (15). Consequently, when a pig organ is transplanted into a human or a NHP, the pre-existing anti-Gal antibodies bind to α-Gal epitopes present on the graft’s vascular endothelium, and induce complement component 3b (C3b) production, complements activation (16), and formation of a membrane attacking complex (MAC). These reactions cause endothelial cells lysis, destruction of the vasculature, and ultimately, graft rejection (17, 18). The loss of endothelial vascular integrity further leads to interstitial haemorrhage, tissue ischemia, and necrosis (19, 20). Moreover, capillaries thrombotic occlusion, fibrinoid necrosis of arterial walls, and neutrophils accumulation contribute to graft failure (21). Nitric oxide species (NOS), reactive oxygen species (ROS), and other free radicals are also key components of the rejection process. The histopathological features of HAR characterize by disruption of vascular integrity, oedema, fibrin-platelet rich thrombi, and interstitial haemorrhage with widespread deposition of immunoglobulins and terminal complement products on vessel walls (21, 22).

Two ways are usually taken to prevent HAR. One consists in knocking out the *α1,3GT* gene in pigs (GTKO pigs) (23, 24), while the other relies on inhibiting complement activation by inducing the expression of human complement-regulatory proteins, i.e., hCD46, hCD55, and hCD59, on pig cells (25). Kuwaki et al. reported that the elimination of the α-Gal epitopes successfully averted HAR in baboons receiving hearts from GTKO pigs (n = 8) and increased the pig heart survival by 2–6 months (median, 78 d) (26). In a study using pig liver transplant to NHPs, genetically engineered expression of hCD55 or of hCD46 combined with GTKO was associated with a survival time of seven to nine days while the wild-type (WT) liver graft did not extend over three days (27, 28). Combination with GTKO, hCD46 or hCD55 expression reduced the early graft failure (signs of early transplantation failure within three days of transplantation) to 7% compared to 43% with GTKO organs (29).

Although these measures allow the grafts to survive beyond 24 hours, graft failure can still result from antibody recovery, through a mechanism termed acute humoral xenograft rejection (AHXR), acute vascular rejection, or delayed xenograft rejection (DXR), as explained below (20).

### 2.2 Delayed xenograft rejection

Delayed xenograft rejection refers to post-HAR and is also called acute humoral xenograft rejection from a mechanistic prospect, or acute vascular xenograft rejection (AVXR) from a pathophysiological prospect. AHXR or AVXR define xenograft injuries occurring within the vasculature and involving antibodies, while complements play a minor role during this type of rejection (21). In other articles, DXR may refer to antibody- and complement-independent cellular xenograft rejection (CXR) (30). Although there are clear differences between the mechanisms underlying these different terms, these are often used interchangeably and there is no international standard. Some efforts should be considered to achieve internationally recognized definitions and descriptions of these different concepts. Below, we discuss the concepts of AHXR and CXR in separate sections.

#### 2.2.1 Acute humoral xenograft rejection

**Figure 2:**
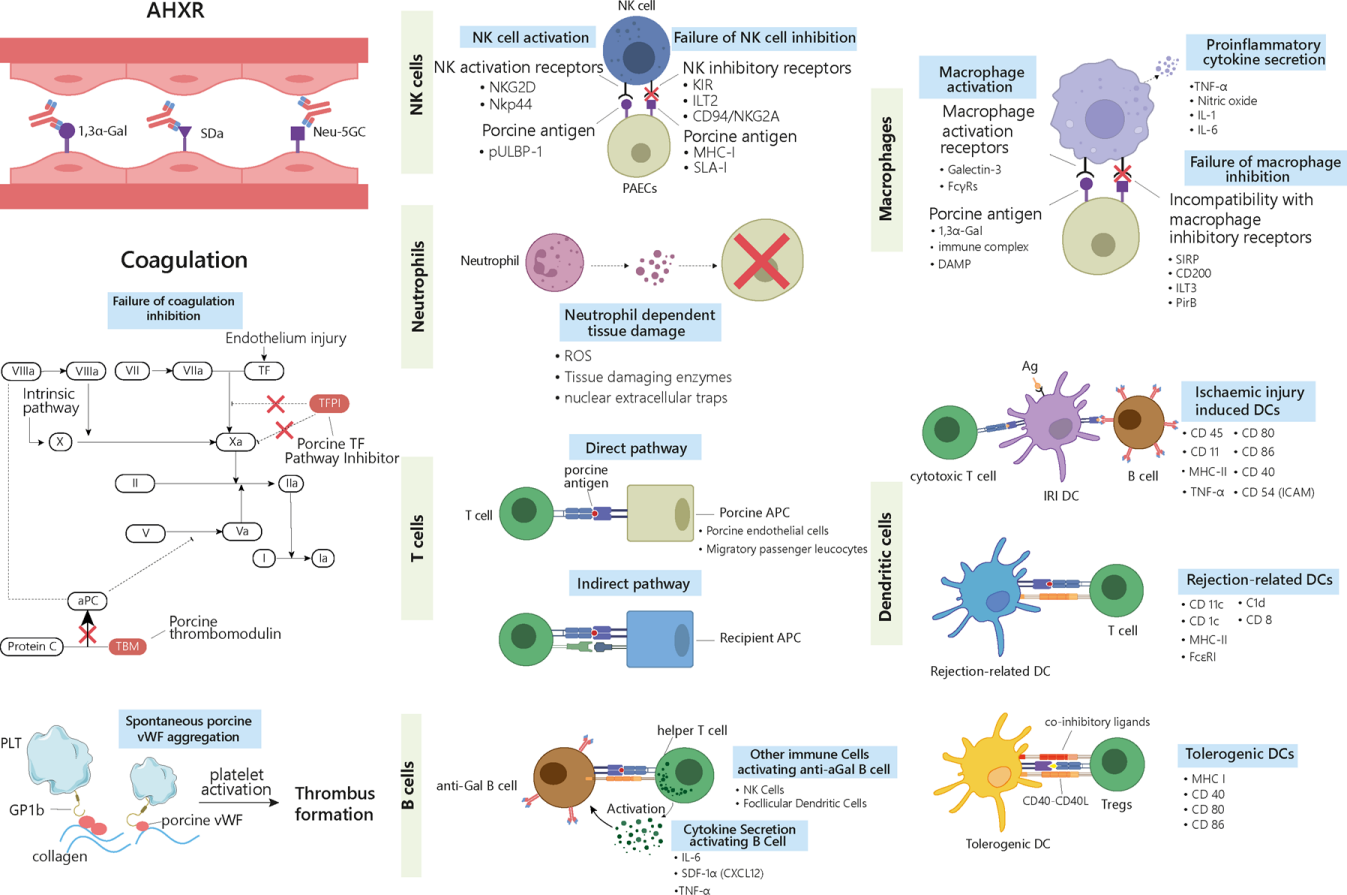
Delayed xenograft rejection: AHXR, cellular xenograft rejection and coagulation dysregulation. The antibodies involved in AHXR are mainly directed against 1,3α-Gal epitopes and non-Gal epitopes such as Neu-5GC and SDa blood group. During cellular responses, ligands on the graft cells activate recipients’ NK cells and macrophages *via* different activating receptors. Meanwhile, graft cells fail to deliver inhibitory signals to the activated cells and enhance their proinflammatory and cytotoxic properties. Neutrophils can generate ROS, tissue-digesting enzyme, and NETs upon activation by graft cells, which causes tissue damage. Three types of DCs are involved in graft rejection. IRI-related DCs activate both cytotoxic T cells and B cells, thus triggering both cell- and antibody-mediated rejection. Rejection-related DCs promote acute and chronic rejection by activating T cells. Tolerogenic DCs suppress immune rejection by inducing Tregs. The different surface markers characterizing the different immune cells are listed in the right-hand side of the cells. T cells are involved in rejection through both direct and indirect pathways. In the direct pathway, porcine APCs present antigens and activate host’s T cells. In the indirect pathway, the graft antigens are presented by the host’s APCs. B cells are activated by T helper cells and secreted cytokines. Antibodies released by activated B cells that have differentiated into plasma cells contribute to graft rejection. AHXR and cellular xenograft rejection are both accompanied with coagulation dysregulation, where porcine TFPI cannot fully inhibit factor Xa and fails to inactivate TF. Porcine TBM also fails to regulate protein C. Porcine vWF can aggregate spontaneously and activate the host’s platelets through GP1b receptors. Altogether these reactions lead to the formation of thrombus in the graft vessels. AHXR, acute humoral xenograft rejection; Ag, antigen; aPC, activate protein C; DC, dendritic cell; IRI: ischemia-reperfusion injury; NET, nuclear extracellular traps; TBM, thrombomodulin; TF, tissue factor; Tregs, T regulatory cells; vWF, von Willebrand Factor.

Provided that a xenograft does not fail due to HAR, a second step consist in overcoming AHXR, which causes immunological destruction within a few days to a few weeks (**Figure 1B**) (14). The histological characteristics of AHXR are focal ischemia and diffuse intravascular coagulation mediated by both humoral and cellar immune responses provoking endothelial cell activation and exaggerated inflammation (31, 32). Lin et al. reported that anti-Gal antibodies removal from the blood of baboons prevents AHXR of porcine organs transgenic for human decay-accelerating factor and CD59, demonstrating that Gal-specific antibodies were implicated in AHXR, in addition to HAR (33). However, antibody-meditated rejection still occurred during transplantation of GTKO pigs’ kidneys or hearts into NHPs, and eventually led to graft failure over the course of several days (34, 35). These results suggest that non-Gal antigens also contribute to AHXR (34, 35).The presence in the recipient of pre-existing antibodies against non-Gal epitopes, such as carbohydrate N-glycolyl neuraminic sialic acid (Neu5Gc), glycan SDa, defining the blood group of the same name, was also implicated in rejection (**Figure 2**) (36–38). Zhu et al. identified Neu5Gc as a crucial non-Gal xenoantigen in 2002 (36), while the SDa blood group was discovered in 1967 (39). Neu5Ac is hydroxylated to produce Neu5Gc by the cytidine monophosphate-N-acetylneuraminic acid hydroxylase (CMAH) present in pigs and other animals, but not in humans (40). The gene encoding this enzyme has been inactivated during primate evolution (41). Double knockout (DKO) of the genes responsible for Gal and NeuGc synthesis in mouse reduced significantly xenoreactive antibody-mediated complement-dependent cytotoxicity of human sera towards mouse tissues, compared to wild-type mouse tissues. The gene encoding the Beta-1,4-N-acetyl-galactosaminyltransferase 2 (β4GALNT2) is responsible for the SDa positive blood group. Its inactivation can significantly diminish porcine xenoantigenicity and reduce the effects of human and NHP non-Gal antibodies (42). Therefore, Neu5Gc and SDa represent key targets for clinical xenotransplantation. Other non-Gal antigens such as Gabarapl1 (GABA type A receptor-associated protein like1), and COX-2 can also induce antibody-mediated rejection (43). In addition, shared common epitopes of human leucocyte antigen (HLA) and swine leucocyte antigen class I (SLA-I) could lead to cross-reactivity between human and porcine (44). Some studies showed that human CD8+T cells were capable of recognizing SLA-I and elicited immune responses and anti-HLA class II antibodies in patients could cross-react with SLA-II (45–48). Immunoengineering of the vascular endothelium to silence SLA expression might be feasible to reduce the immunogenicity (49–52).

The binding IgM and IgG to non-Gal antigens triggers the onset of AHXR by activating the complement cascade, which is a key actor of antibody-mediated damages (53). Besides, humoral responses against endothelial epitopes can also cause activation and damage of the vascular endothelium through other mechanisms, for example antibody-dependent cell-mediated cytotoxicity (ADCC) and inflammation. Further, neutrophils may activate porcine endothelial cells (pECs) (54). Natural killer (NK) cells (55) and macrophages (56) are also agents of AHXR, but the exact pathogenesis involving these cells remains unclear. The histological characteristics of AHXR include extensive interstitial bleeding, infarction, necrosis, thrombosis, neutrophil infiltration, and massive deposition of immunoglobins, complements, fibrin and platelets (34). These manifestations are similar to those found in HAR (14).

Genome editing tools, such as zinc-finger nucleases, transcription activator-like effector nucleases (TALEN), and CRISPR (clustered, regularly interspaced, short palindromic repeats)/Cas9 (CRISPR-associated protein 9), have paved the way for significant breakthroughs in dealing with AXHR by creating DKO (*GGTA1/CMAH* or *GGTA1/B4GALNT2*) pigs, or triple knockout (TKO, *GGTA1/CMAH/B4GALNT2*) pigs, which had been first generated in the USA (57–60). Cells from *GGTA1/CMAH* pigs were associated with reduced affinity of the human antibodies compared with cells from GTKO pigs (59). Moreover, the absence of Neu5Gc and Gal epitopes from murine xenogeneic cells has been proven to dampen the immune reaction provoked by the pre-formed antibodies contained in human serum (61). These data suggest that the deletion of the Neu5Gc epitopes could help increase the survival time of xenografts. *In vitro* evidence showed that the reactivity of pre-existing antibodies against the xenografts could be reduced by inactivating the *B4GalNT2* gene (60). This result indicates that TKO pig organs can promote major development for transplantation into human compared with GTKO and DKO xenografts, but still TKO pig organs were not sufficient to achieve prolonged graft survival, limiting by complement-mediated coagulopathies (62). It should also be noted that a fourth xenoantigen seems to be exposed when the Neu5Gc epitopes are absent, and that baboons and other Old World monkeys are more likely to reject this fourth xenoantigen than humans are (63). Therefore, TKO pig heart or kidney transplantation in baboons may appear more problematic than they would be in a clinical trial involving humans. Due to the cross-reactivity between HLA-specific antibodies and SLA, modifying SLA genes may help further reduce the human immunoglobulin binding against pig cells. Two inactivating mutational approaches targeting either the light β-chain or a conserved region in the heavy α-chain of SLA-I have been successfully utilized in the pig genome to generate pigs with no or limited expression of SLA (45). PBMC from these pigs induced almost no proliferation of purified human CD8+ T cells (45). However, complete removal of SLA in pig genomes may not be an ideal solution due to logistical, immunological, and infectious consequences of SLA deletion (64). Instead, for highly HLA sensitive recipients, an ideal organ-source pig might be one with site-specific mutations to eliminate cross-reacting antibody binding.

#### 2.2.2 Cellular xenograft rejection (acute cellular rejection)

If both HAR and AHXR are overcome, but immunosuppressive therapy is insufficient, CXR may occur and lead to graft rejection within days or weeks following transplantation (65, 66). CXR can be mediated by the innate and/or the adaptive immune system, and may involve NK cells, macrophages, neutrophils, dendritic cells (DCs), T cells, and B cells (**Figure 2**).

##### 2.2.2.1 NK cells

NK cells mediate xenograft rejection by direct NK cell cytotoxicity (NKCC) or ADCC. Upon direct contact with the target cells, NK cells deploy cytotoxic functions by engaging a series of activating and inhibitory receptors and ligands (53). Recent research has revealed a mechanism whereby NK cells adhere to and transmigrate through the porcine endothelium by interacting with an as yet to define porcine ligand, *via* CD49, integrins including CD11a/CD18 and CD11b/CD18, and CD99 (67). NKG2D and NKp44 are activating receptors that can bind porcine pULBP-1 and an unidentified ligand, respectively (68). Their engagement initiates the release of lytic granules containing cytotoxic proteins such as perforin and serine esterase, which lead to the lysis of the donor’s endothelial cells (69). Inhibitory receptors such as killer-cell immunoglobulin receptors (KIRs), immunoglobulin-like transcript 2 (ILT2), and CD94/NKG2A heterodimers mainly recognize MHC-I molecules (70, 71). Low expression or absence of MHC-I molecules on porcine cells leads to reduced inhibitory signals transduced to the NK cells, which triggers their activation and subsequent lysis of the porcine cells (72, 73). Stable expression of transgenic human leukocyte antigen (HLA)-Cw3 and/or G, and/or E, on porcine cells could protect the xenograft from human NK cytotoxicity (74).

Besides direct cytotoxicity, NK cells can also employ complement-independent ADCC to destroy the graft (75). Preformed natural antibodies against α-Gal carbohydrates or Neu5Gc can bind to the pECs with their Fab portion. Fc receptors (FcRs), including CD16 (FcγRIIIa) on NK cells, recognize the Fc portion of the antibodies, triggering a signalling cascade that causes degranulation (76). In addition, CD16 recognizes induced antibodies against SLA-I, which can mediate ADCC (77). Furthermore, NK cells can promote the production of non-Gal antibodies against the graft in a T-independent manner, by interacting with splenic marginal zone B cells *via* CD40/CD154 interaction (78).

The role of NK cells in CXR needs further elucidation to prolong xenotransplant survival. To date, most knowledge comes from *in vitro* studies and animal models. Future *in vivo* studies on pig-to-NHP transplants are needed to clarify the role of NK cells in xenograft rejection.

##### 2.2.2.2 Macrophages

Macrophages carry out diverse functions, ranging from phagocytosis, cytokine production, antigen presentation, to tissue repair. Like for other innate immune cells, Toll-like receptors on the surface of the macrophages can recognize nonself molecules such as danger-associated molecular patterns (DAMPs) arising from injured xenogeneic cells (79–82), pathogen-associated molecular patterns (PAMPs), polysaccharides, and polynucleotides (83). Under the synergistic effect of Toll-like receptors activation and interferon γ (IFN-γ), macrophages are licensed to process and present xenoantigens, promote the differentiation of pro-inflammatory T helper 1 (Th1) and T helper 17 (Th17) by producing interleukin-12 (IL-12), and exert direct cytotoxicity by producing proinflammatory cytokines such as tumour necrosis factor α (TNFα), IL-1, IL-6, and nitric oxide (84, 85). Interspecies incompatibility of CD47 was also reported to contribute significantly to macrophage-mediated rejection of xenogeneic cells. That is, in a human macrophage-like cell line, porcine CD47 does not stimulate the inhibitory receptor signal-regulatory protein α (SIRPα), while soluble human CD47-Fc fusion protein does, which inhibits porcine cell phagocytosis (86). Human macrophages were found to phagocytose porcine red blood cells independently of the presence of antibodies or complement activation, even in setups where the α-Gal epitopes were absent from the porcine cells (87).

In CXR, macrophages mainly act by promoting T-cells mediated rejection (88–90). Feng et al. used IL-10/Fc to treat mice before or after transplantation of a pancreatic islet xenograft, and found that the T cell effector functions were inhibited, with reduced IFN-γ and IL-4 expression probably due to the inhibition of IL-12 production by macrophages (91). In whole organ xenografts, Lin et al. showed that the macrophages might be the cause of rejections occurring within 3–6 days. In their study, hamster hearts were transplanted into genetically engineered T cell-deficient rats depleted or not of NK cells, which demonstrated that in absent of T and NK cells the graft was still rejected. The spleen of the recipient and the rejected organs were predominantly infiltrated by macrophages (92). Since abundant evidence shows that macrophages play a role in xenograft rejection, the regulation of their activity might enhance the survival of future xenografts.

##### 2.2.2.3 Neutrophils

There are at least three mechanisms whereby activated neutrophils can induce tissue damage: (i) ROS generation; (ii) release of tissue-digesting enzymes; and (iii) nuclear extracellular traps (NETs) formation (93).

Human neutrophils can directly recognize pECs, and subsequently upregulate adhesion molecules and render porcine cells more vulnerable by exposing them to NK cell-mediated cell lysis (94). Specific recognition pathways were thought to be responsible for the adhesion of human neutrophils to porcine endothelium, as under flow conditions, adhesion occurred independently of the presence of α-Gal or ICAM-1 (95). Mohanna et al. demonstrated that neutrophils can be activated directly by porcine aortic endothelial cells, which subsequently causes a transient rise of calcium flux triggering the production of reactive oxygen metabolites and inflammatory cytokines (54). Cardozo et al. further demonstrated that the adhesion of human neutrophils to pECs can be facilitated by a soluble chemotactic factor produced by pECs (96). Activated neutrophils also exert cellular damages by generating superoxides *via* NADPH oxidase activity (97).

Apart from ROS generation, leukocyte proteases have been implicated in neutrophil-mediated graft tissue damage, through disruption of endothelial cell junctional complexes (98). For instance, after acute ischemia/reperfusion insults in liver, neutrophil elastase (NE) breaks down graft’s homeostatic barriers by degrading the extracellular matrix (ECM) components, including collagen, elastin, and fibronectin (99).

“NETosis”, a program for production of neutrophil extracellular traps (NETs), is a unique process whereby neutrophils induce inflammation and cell death in porcine grafts. NETs are mainly composed of antibacterial peptides, histones, and serine proteases that accumulate in the lung in both experimental and clinical primary graft dysfunction (PGD). Disruption of NETs with DNase I reduces lung injury (100).

##### 2.2.2.4 Dendritic cells

The most efficient antigen presenting cells (APCs) in activating T cells and initiating immune tolerance are the DCs. Based on different surface markers, DCs are generally divided into three subsets: (i) DCs involved in ischemia-reperfusion injury (IRI) and expressing C1d, CD8α, CD11c, CD40, CD45, CD54 (ICAM), CD80, CD86, MHC-II, and TNFα, but are negative for CD4 and CD205 (101); when activated by antigens released during ischemia-reperfusion, these DCs can trigger both cellular and antibody-mediated rejection, resulting in harmful antibodies secretion by activated B cells and killing of donor cells by cytotoxic T cells (102); (ii) rejection-related DCs, promoting acute and chronic rejection *via* different interactions with T cells and are characterized by the expression of CD11c, MHC-II, CD1c and FcϵRI (103); (iii) tolerogenic DCs, suppressing the rejection process by dampening the T cell effector functions and promoting T regulatory cells (Tregs) activity; these DCs express significantly lower levels of MHC, T cell co-stimulatory molecules, such as CD40, and CD80/86, and inhibitory ligands, such as programmed death ligand-1 (PD-L1) and death-inducing ligands, reflecting their non-phagocytic profile (104, 105). In response to specific signals such as DAMPs, host DCs can acquire a rejection-related phenotype, which can further evolve towards a tolerogenic phenotype upon treatment with rapamycin, IL-10, vitamin D, or low-dose granulocyte-macrophage colony-stimulation factor (GM-CSF). Tolerogenic DCs reduce CD4+ T cell activation and impair CD8+ T cell functions, which helps suppressing graft rejection (102). Manna et al. reported that DCs activation by the porcine aortic endothelial cells could be blocked by a pre-treatment of the DCs with antibodies specific for the human leukocyte function-associated antigen-1 or CD54 (106). However, the exact mechanisms underlying DCs participation to the rejection process still need to be clarified in order to develop anti-rejection drugs.

##### 2.2.2.5 T cells

The role of T cells in cellular rejection during pig-to-baboon xenotransplantation has been demonstrated, although the relevant studies were only few (107, 108). Upon xenotransplantation, T cells are activated by both direct and indirect pathways (109). In the direct pathway, porcine APCs expressing CD80/86 constitutively, such as pECs and migratory passenger leukocytes, can directly prime primate T cells (110). Simultaneously, primate TCRs interact with SLA-I/II peptide complexes, resulting in T cell-mediated cytotoxicity against the porcine vascular endothelium. In the indirect pathway, T cell activation occurs through the presentation of porcine peptides by the hosts’ APCs. T cell activation requires antigen recognition through the TCR coupled with costimulatory signals (111), involving CD40-CD154 and/or CD80/CD86-CD28 interactions (112). Different drugs targeting these costimulatory pathways could be administrated in xenotransplantation, as for example anti-CD40 mAb and CTLA4Ig, as discussed below. In addition, abrogation of SLA-I expression has been proved to silence T-cell and NK cell-mediated cell lysis (113, 114).

##### 2.2.2.6 B cells

One percent of total circulating IgGs, and 1–4% of total IgM in the human serum are directed against α-Gal epitopes (115). Previous studies established that the cells producing anti-Gal antibodies reside mainly in the spleen, and to a lesser degree in lymph nodes and bone marrow (116). This location matches that of a recently described splenic B cell subtype characterized by a Mac1+ B1b-like phenotype (117). Antibody production results from the interaction between B cells and other immune cells, including T cells, NK cells, and follicular DCs. Immunization of α1,3GT KO mice with pig cell membranes induces clonal expansion of anti-Gal B cells that can present antigen to T helper lymphocytes *via* MHC-II and provide CD40 co-stimulation, causing cytokine production by the activated CD4+ T cells. This process provides activated B cells with the helper signals necessary to their proliferation and maturation in germinal centres, resulting in production of high-affinity anti-Gal antibodies (118). Another study suggested that marginal zone B cells can produce xenoantibodies after receiving help from NK cells, independently of T cell help (78). Moreover, the follicular DCs, expressing the complement receptors 1 and 2, can activate α-Gal-reactive B cells by presenting α-Gal immune complexes (119).

As B cells are the main sources of elicited anti-porcine antibodies, they represent an important target to overcome AHXR. Efficient depletion of circulating and secondary lymphoid organ-resident B cells by anti-CD20 antibody at the time of transplant prevents anti-pig humoral responses and resulting graft injury, and significantly delays or prevents the systemic dysregulation of the coagulation pathway and thrombotic microangiopathy (120, 121). Zhao et al. reported that anti-high mobility group box protein 1 (HMGB1)-neutralizing antibody prolonged xenograft survival, and dampened tissue damage and immune cell infiltration by suppressing xenoreactive B cell responses (122).

#### 2.2.3 Coagulation dysregulation in DXR

Coagulation dysregulation was first described by Ierino in the late 1990s. Both AHXR and cellular xenograft rejection are accompanied with coagulation dysregulation, which results in the development of thrombotic microangiopathy in the graft. Antibody-mediated and cellular rejections cause endothelium injury, exposing tissue factor (TF) and collagen. The binding of TF to activated factor VII (FVIIa) initiates thrombin generation, converting fibrinogen into fibrin. Simultaneously, sub-endothelial collagen triggers the accumulation and activation of platelets (123). Importantly, this process is enhanced by molecular incompatibilities between the primate and porcine coagulation homeostatic systems. The porcine tissue factor pathway inhibitor (TFPI) cannot fully inhibit the factor Xa in primates and fails to inactivate TF (124). In addition, the porcine thrombomodulin (TBM) is unable to regulate the primate thrombin, and thus, fails to activate the protein C (**Figure 2**) (125). Another incompatibility lies between the primate platelet lycoprotein 1b (GP1b) and the porcine von Willebrand Factor (pvWF). pvWF can spontaneously aggregate without shear stress and activate primate platelets through the GP1b receptor (126). The subsequent graft vessel thrombosis caused by fibrin deposition and platelet aggregation eventually leads to ischemic injury (**Figure 2**) (26, 127). Approaches to tackle coagulation dysregulation include the transgenic expression of human complement proteins (hCRP) and coagulation proteins such as human TBM by the donor porcine organs (128).

### 2.3 Chronic rejection

Chronic rejection usually occurs several months to years after organ transplantation. It has similar histopathological characteristics to those found in allotransplantation and are mainly related to thrombotic microangiopathy, characterized by the proliferation of graft vascular endothelial cells, vessel narrowing, interstitial fibrosis, which ultimately, result in graft failure (**Figure 1C**) (129). Since there are only few long-term survivors to xenotransplantation, the mechanism of chronic rejection has not been sufficiently documented, but it is almost certainly related to long-term, low-amplitude immune responses.

Current research indicates that chronic rejection involves both immune and non-immune factors. Molecular incompatibilities between the porcine and the NHP coagulation factors may play a vital role (130). Mohiuddi *et al.* reported that gene-editing of pig heart (*GTKOhTg.hCD46.hTBM*), alongside with anti-CD40 monoclonal antibody treatment, allowed for successful survival of a graft for 236 days in baboons. This result indicates a crucial role of human TBM expression combined with anti-CD40 treatment not only for the long-term survival of the graft, but also to avoid thrombotic microangiopathy and other coagulation-related problems (131). Another study demonstrated that transgenic expression of human CD39 (a major vascular nucleotidase that converts ATP and ADP into AMP, further degraded into anti-thrombotic and anti-inflammatory adenosine) in mouse significantly prevents thrombotic events in the heart graft and improves the duration of graft survival from three days in WT mice, to six days in the transgenic mice (132).

Another study by Kim et al. achieved a long-term survival of 499 days with pig-to-rhesus macaque renal xenografts by depleting CD4+ T cells (46), indicating that these cells are responsible for chronic rejection. Similar to allotransplantation, the host’s MHC class II molecules recognize porcine xenoantigens and present them to the host’s CD4+ T cells, leading to their activation (109). Yet, the mechanisms involving the CD4+ T cells in chronic rejection are poorly understood. Besides, a sustained inflammatory response is still a key challenge to achieve successful grafts. Future studies should explore the roles of inflammatory cytokines such as IL-6, TNF-α, IL-17, and their inhibitors to uncover therapeutic targets (133, 134).

## 3 The prevention of xenotransplantation rejection

**Figure 3:**
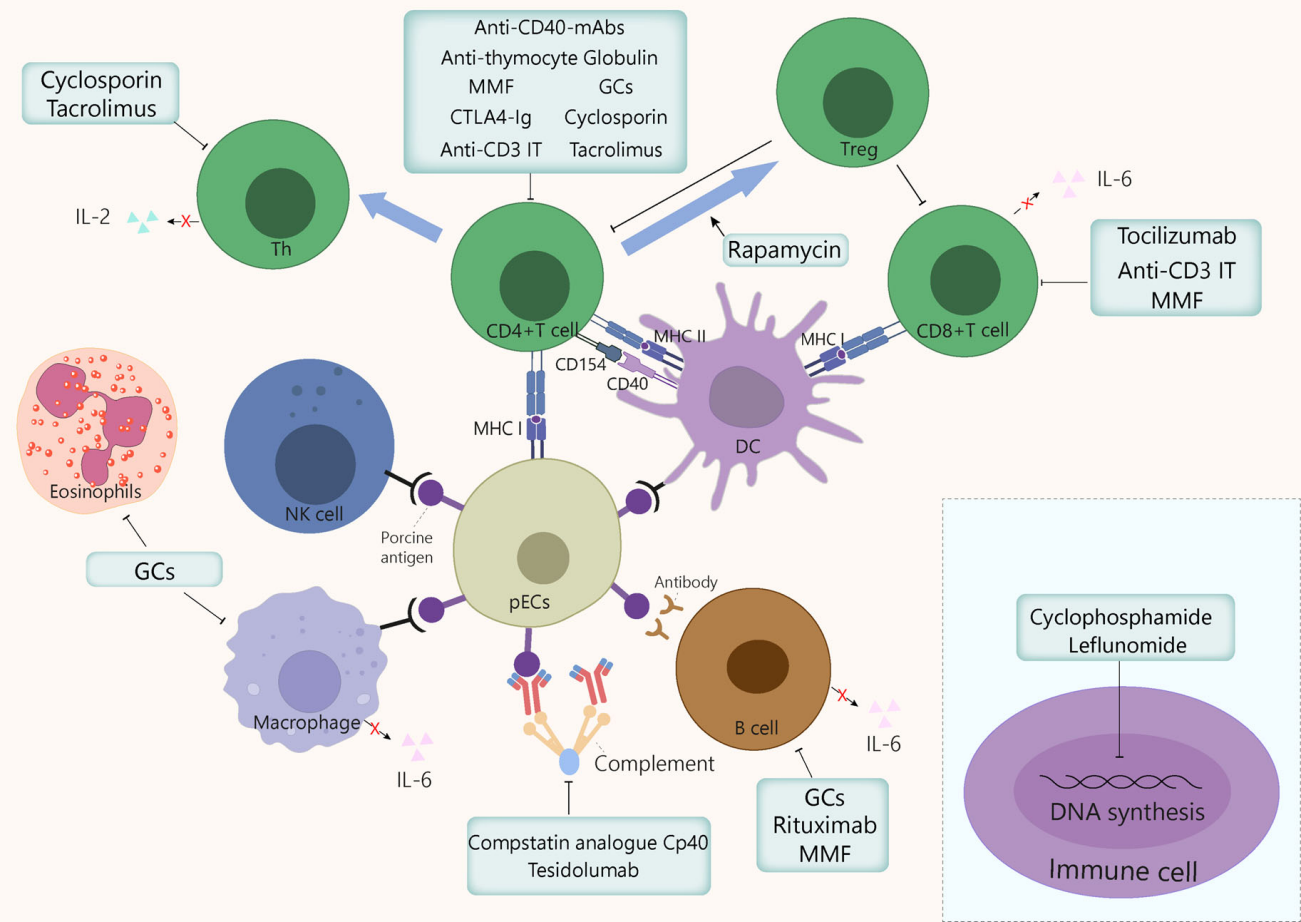
Mechanism of action of the immunosuppressants commonly used in xenotransplantation. GCs exert anti-inflammatory and immunosuppressive effects by inhibiting macrophages, eosinophils, T cells, and to a lesser extent, B cells, by binding to GC receptors in cytoplasm. Cyclosporin binds to cyclophilin, then this drug-immunophilin complex binds to calcineurin, which subsequently prevents Th cell activation and IL-2 production, which eventually inhibits T cell clonal proliferation. Tacrolimus inhibits T cells proliferation by binding to FKBP, which inhibits several transcription factors involved in the production of proinflammatory cytokines. Cyclophosphamide blocks DNA alkylation in various cell types, leading to programmed cell death induction and preventing cell division. Leflunomide inhibits the synthesis of pyrimidines, thus arresting cell cycle in S phase. Mycophenolate mofetil prevents T and B cell proliferation by specifically inhibiting a purine pathway required for lymphocyte division. Polyclonal anti-thymocyte globulins are mainly directed against T cells. However, other immune cells sharing common surface antigens with T cells can also be affected to a lesser extent. Monoclonal antibodies target specific cytokine pathways (e.g., IL-6Rα) or cell surface markers, such as CD3, for anti-C3 IT, or CD20, for rituximab. The IL-6 receptor inhibitor tocilizumab reduces systemic inflammation and inhibits of CD8+ T cell and B cell differentiation. Anti-CD3 IT can deplete CD3+ T cells transiently and reduces the number of T cells in circulation and in lymph nodes. Rituximab is a B cell-depleting drug that targets CD20. Rapamycin exerts immunosuppressive and anti-proliferative effects of T cells by inhibiting the activation of S6K1 and PI3 kinase signalling. CTLA-4Ig and anti-CD40mAb target the costimulatory pathways CD80/86:CD28 and CD154:CD40, respectively, thereby dampening T cell activation. The compstatin analogue Cp40 and Tesidolumab inhibits complement C3 and C5 respectively, thereby reducing complement activities. FKBP, FK506 binding protein; GCs, glucocorticoids; IT, immunotoxin; mAb, monoclonal antibody; MMF, mycophenolate mofetil, pECs, porcine endothelial cells.

Since 2009, porcine models with new genetic modifications have been constantly implemented to improve molecular compatibilities. As gene editing techniques such as zinc finger nucleases, TALEN, and CRIPSR/Cas9 genome editing system improve, the production of multiple-gene edited pigs has become easier and faster (135). This section of the review will focus on the mechanisms and usage of common immunosuppressants in xenotransplantation area (**Figure 3**).

Immunosuppressive agents are used commonly in treatments for transplantation rejection. Conventional immunosuppressive therapy, for example, corticosteroids, tacrolimus, and cyclophosphamide (136–138), when used at high dosages, may delay graft failure. In non-human concordant models for kidney or liver xenografts, long-term survival can be achieved with conventional immunosuppressive agents (139). In 2000, a co-stimulation blockade-based immunosuppressive therapy was introduced to xenotransplantation by Buhler et al. (140), and has proven more successful than common therapies.

### 3.1 Glucocorticoids

Glucocorticoids (GCs) belong to the steroid family and were discovered in the 1940s (141). GCs have been used as first-line medication during the induction and maintenance phases after transplantation to prevent acute rejection. In pig-to-primate xenotransplantation, immunosuppressive GC regimens have also been used in most trials (142–144). In an islet xenotransplantation study, all monkeys experienced a normalization of all diabetic and glycemic parameters within four days of GCs administration, suggesting a reversal of diabetes mellitus (145). In another pre-clinical study, the longest survival time (78 days) for a porcine renal xenograft was obtained by applying GCs combined with other immunosuppressants (146).

The 17-hydroxy, 21-carbon steroid configuration characterizing the GC molecules is required for their activity and binding to the GC receptors (GCRs). Changes in this configuration can alter the pharmacodynamic specificities of the GCs (147). GCs’ major anti-inflammatory effects result from interferences between activated GCRs and proinflammatory transcription factors such as nuclear factor-κB and activator protein-1 (148). The immunosuppressive mechanisms triggered by GCs include: (i) T cell depletion *via* inhibition of IL-2, which prevents Th1 differentiation and subsequently results in T cell apoptosis; (ii) prevention of B cell clonal expansion through inhibition of the production of IL-2 and related peptides, which reduces antibody production; (iii) induction of eosinophil apoptosis either directly or through IL-5 inhibition (147, 149); (iv) downregulation of the Fc receptors and MHC class II molecules on macrophage surface *via* proinflammatory cytokine inhibition, e.g., IL-1 and TNF-α, and prostaglandins (150); (v) widespread decrease of inflammatory responses in the host by induction of lipocortin-1 (annexin-1) synthesis; (vi) acceleration of lymphocytes apoptosis and abrogation of alloimmune responses to third-party antigens, such as allergens and autoantigens. In conclusion, all immune cells that express GCRs can be modulated by GCs, and as a result, lose abilities such as migration or phagocytosis.

The exact dosage of GCs for induction and maintenance phases varies between different institutions. Methylprednisolone doses vary from 1 to 15 mg/kg, for a duration varying from four days to continuous treatment (143, 146). A metanalysis showed that long-term GCs usage may increase the risk of infections for the hosts (151). Pulse dose steroids are now favoured for treating acute allograft rejection but are less crucial in maintenance immunosuppressive therapy. According to some guidelines for clinical practice, GCs could be discontinued early at first week post transplantation for patients with low immunological risk who receive depleting antibodies as induction therapy (152).

### 3.2 Calcineurin inhibitors

Two common calcineurin inhibitors (CNIs), cyclosporin and tacrolimus, inhibit the dephosphorylation of nuclear factor of activated T cells (NFAT) by calcineurin, preventing its nuclear translocation and subsequent calcineurin-dependent gene transcription (153, 154). This inhibition results in decreased T cell maturation and lymphokine production, including that of IL-2.

#### 3.2.1 Cyclosporin

Cyclosporin is a cyclic polypeptide consisting of 11 amino acids, most of which are hydrophobic. The discovery of cyclosporin in the early 1980s had a huge impact on the transplantation field by decreasing drastically the rate of acute rejection. This drug binds to cyclophilin; the drug-immunophilin complex then binds to calcineurin, which prevents T cell activation and IL-2 production, thus inhibiting T cell clonal proliferation. Cardiac xenografts treated with steroids and cyclosporin achieved a survival of 77 days, with no signs of hyperacute rejection or cyclosporin-induced malignancies (155). In baboon-to-monkey liver xenotransplantation, two monkeys survived for 91 and 1076 days, respectively, with cyclosporin administered after transplantation, for example, at doses of 3 to 8 mg/kg/day (139), or 20 mg/kg intravenously two hour before transplantation followed by oral administration on next three days (143). In a cardiac xenograft transplantation, the highest cyclosporin blood levels (around 1000 ng/ml) correlated with the highest graft survival rate of the host animals (142).

#### 3.2.2 Tacrolimus

Tacrolimus, a 23-membered macrolide lactone, was isolated for the first in 1987 from *Streptomyces tsukubaensis* (156). It inhibits T cell proliferation by binding FK506 binding protein (FKBP) (157), which inhibits calcineurin by binding it specifically and competitively (158). Subsequently, NFAT nuclear translocation is inhibited, which provokes the downregulation of downstream genes encoding cytokines, including TNF-α, IL-2, IL-3, IL-4, CD40L, IFNγ, and GM-CSF (158, 159). This tacrolimus-induced cascade finally leads to reduced T cell proliferation. Tacrolimus was first approved for liver transplantation in 1994, and since, its used has been extended to become the backbone of immunosuppressive therapy after solid organ transplantation. Later, it has been used for induction and maintenance immunosuppressive therapy, usually in combination with GCs that are then rapidly de-escalated (160). Tacrolimus effectively prevents acute rejection and leads to lower rejection rates and longer rejection-free periods (161, 162). In pig-to-rat islet xenotransplantation model, tacrolimus also exerts a noticeable immunosuppressive effect (163, 164). The oral bioavailability of tacrolimus ranges from 5 to 67% (mean value of 27%), and its half-life ranges from 3.5 to 40.5 hours (165). Protocols with tacrolimus constantly evolve (166). In a pre-clinical study on pig-to-NHP islet xenotransplantation, tacrolimus was orally administered daily from day −3 to up to day 56 to achieve stable levels (3–6 ng/mL) (164). In another study, tacrolimus was injected intramuscularly twice daily at a dose of 0.05 mg/kg from day –2 and for up to 6 months after transplantation (167). The preferred administration route remains oral rather than sublingual or intramuscular (168). The adverse effects associated with this drug are mainly nephrotoxicity and neurotoxicity, which can be mild to severe. To avoid adverse effects, it is important to maintain tacrolimus at a stable dose. Because the *CYP3A5* genotype is associated with a remarkable impact on tacrolimus pharmacokinetics, it should be considered in the dosing algorithm of this drug (169). Moreover, some research attempted to develop machine-learning models to predict tacrolimus dose stability, which might provide more accurate approaches to achieve personalized medicine in clinics (170).

### 3.3 Antiproliferative agents

#### 3.3.1 Cyclophosphamide

Cyclophosphamide (CYC) is widely used to prevent transplant rejection and graft-vs-host complications (171). It is a nitrogen mustard drug that affects DNA alkylation in a non-cell cycle phase-specific manner, and is toxic for all human cells to various degrees (172). The active form of CYC inhibits protein synthesis *via* DNA and RNA crosslinking, leading to programmed cell death and prevention of cell division (173).

The immunosuppressive effect of CYC mainly relies on direct deletion of the host’s mature T cells that are highly proliferating and reactive to the donor’s antigens (174). It also has the capacity to deplete Tregs to counteract immunosuppression in cancer, decrease the production of T cell growth factors, e.g., type I interferons, and precondition host T cells for donor cells, hence attenuating rejection (175). Regimens with CYC in xenotransplantation have proven effective in some cases (146, 176). In pig-to-rhesus corneal transplantation, intravenous injection of CYC followed by pig bone marrow cell transplantation reduced inflammatory cell infiltration (177). CYC is applied typically as a continuous treatment administered orally or intravenously in pulses, with doses ranging from 10 to 40 mg/kg (146, 178). Intermittent intravenous rather than daily oral CYC has been used to minimise bladder and gonadal toxicity. Another side effect of CYC is myelosuppression, which causes leukopenia and neutropenia and can lead to severe and sometimes fatal infections, including viral infections (179).

#### 3.3.2 Mammalian target of rapamycin inhibitors

The mammalian target of rapamycin (mTOR) signalling pathway has important functions in cell growth and metabolism regulation (180). Rapamycin can bind a 12-kDa FK506-binding protein (FKBP12) to form a gain-of-function complex that acts as an allosteric inhibitor of mammalian TOR complex 1 (mTORC1) (181). Rapamycin exerts its immunosuppressive and anti-proliferative properties *via* the inhibition of S6K1, a serine/threonine kinase activated by a variety of agonists (182, 183). In rat-to-mouse islet transplantation, rapamycin could induce Treg-mediated tolerance (184). Moreover, Singh et al. verified that in baboons, treatment with rapamycin increases CD4+ Tregs induction from naïve CD4+ T cells, thereby suppressing anti-porcine xenogeneic response *in vitro* (185). Furthermore, in both allo- and xenotransplantation, graft recipients treated with IL-17-neutralizing antibodies showed the highest percentage of Tregs (186, 187). An example of reported schedule for treatment with rapamycin consists of 0.2 mg/kg during the first three days post-transplantation, followed by treatment every other day until day 14 (184).

#### 3.3.3 Leflunomide

Leflunomide inhibits the dihydro-orotate dehydrogenase, a critical rate-limiting enzyme for pyrimidine synthesis. Therefore, it arrests cell cycle progression from S to G2 phase (188). The literature regarding the role of leflunomide in xenotransplantation is limited but indicates that this drug inhibits rat-to-mouse cardiac xenograft rejection by supressing NF-κB signalling pathway and adaptive immune responses (189).

#### 3.3.4 Mycophenolate mofetil

Mycophenolate mofetil is the semisynthetic morpholinoethyl ester of mycophenolate acid, which prevents T and B cell proliferation by specifically inhibiting a purine pathway required for lymphocyte division (190). MMF, usually administered intravenously at a dose of 20mg/kg twice per day, has been mainly applied together with other immunosuppressants as maintenance regimen and achieved considerable long-term survival of xenograft (the longest reported to be 945 days) in cardiac xenotransplantation (191–194).

### 3.4 Monoclonal or polyclonal antibodies

Monoclonal antibodies (mAbs) are widely used in clinics and experiments. Most have cell-specific immune-modulatory properties directed for example at CD3+ T cells, which are particularly pathogenic in the context of solid organ transplant rejection (195). Recent studies suggested that in the absence of irradiation or chronic immunosuppressive drugs, renal tolerance can be stably established in primates (18–20) by using an anti-CD3 immunotoxins (ITs) that ablate T cells transiently (145, 196, 197). T cell numbers in blood and lymph nodes could be reduced to 1% of their initial values following anti-CD3 IT depletion, which established long-term tolerance towards mismatched renal allografts (145). Anti-CD3 ITs administered two hours pre-transplantation at a dose of 100 μg/kg and again on the day following transplantation has demonstrated efficacy (143). However, this schedule should be further verified in the future.

Subsequently, monoclonal antibodies were generated against specific cytokines that play a role in immune cell-mediated toxicity and tissue damage. IL-6 is induced by inflammation and contributes to CD8+ T cell and B cell differentiation (198). In addition, it is a crucial factor in systemic inflammation and endothelial cell survival after xenotransplantation. More recently, Zhao et al. proposed that IL-6 may promote coagulation and inflammation during xenotransplantation (198). Furthermore, Ezzelarab et al. uncovered that biologics inhibiting the IL-6 pathway (e.g., Tocilizumab) could mitigate systemic inflammation in xenograft recipients (SIXR) and may be required to prevent coagulation dysregulation after xenotransplantation (199). Tocilizumab is a biological that blocks human IL-6Rα, and has been considered to reduce inflammation by inactivating the STAT3 pathway acting downstream of IL-6Rα. Tocilizumab was also found to delay the revascularisation of xeno-islets in a pig-to-NHP model (200). Another case report brought exciting results on the use of tocilizumab in combination with other immunosuppressants, which allowed to achieve a 136 day-pig kidney survival (201). However, another recent research by Zhang et al. reported that serum IL-6 increased in baboons receiving tocilizumab before xenotransplantation. This increase could be detrimental to the survival of the pig xenograft by promoting IL-6 binding to pig IL-6R and subsequent pig cell activation (202). Thus, more clinical trials are needed to determine whether tocilizumab is beneficial or detrimental to xenotransplantation. The dose of tocilizumab was consistently 10 mg/kg in all reported cases, with a treatment schedule usually starting on days -1, 7 and 14, followed by administration every two weeks (198).

Another monoclonal antibody used in transplantation is the chimeric anti-CD20mAb rituximab that leads to B cell depletion (203). In addition of being an effective treatment for post-transplant lymphoproliferative disorders, Rituximab can serve as treatment for acute rejection, as some evidence suggested that it could stop the progression towards chronic antibody-mediated rejection (204). Its mechanism of action may be explained by its impact on B cell modulation of the T cell responses, and its long-term effects on plasma cell development (205). In combination with other immunosuppressants, anti-CD20mAb was reported to achieve a 136 day-pig kidney survival (201). An example of reported schedule for treatment with rituximab in transplantation is 19 mg/kg at days -14, -7, 0 and 7 (206).

Polyclonal anti-thymocyte globulins (ATGs) are antibodies obtained by injecting animals, usually rabbits, with human lymphoid cells such as B lymphoblasts, peripheral T cells, or thymocytes, and then harvesting and processing the sera to purified the immunoglobulins (207). ATGs are predominantly directed against T cells, but other immune cells sharing surface antigens with T cells can also be targeted to lesser degrees, as for example B cells, monocytes, and neutrophils. ATG primary mechanism of action consist in promoting lymphocyte depletion through T cell activation-induced apoptosis and complement-dependent lysis (208). In a preclinical study, ATGs were shown to improve engraftment and survival of neonatal porcine xenoislets (209). They can also extend pig kidney survival when combined with other immunosuppressants (201). ATGs are usually prescribed before transplantation at a dose of 10 mg/kg on day –3 (201), or at a dose of 5 mg/kg on days –2 and –1 (206).

### 3.5 Blockade of costimulatory signals

#### 3.5.1 Blockade of CD80/86:CD28 costimulatory pathway by CTLA4Ig

T cell activation requires co-stimulation *via* engagement of CD28 on the T cell with CD80/86 on the APC. Cytotoxic T lymphocyte–associated protein 4 (CTLA4) is a competitive inhibitor of CD80/86 that downregulates T cell responses (210). This T cell suppressive activity served to engineer a human IgG heavy chains coupled with CTLA4 to create a fusion antibody able to prevent graft rejection (211). The new generation CTLA4Ig, belatacept, displayed a significantly higher affinity for CD80/86 in a pre-clinical renal transplantation model in primate (212), and showed greater efficacy in modulating adaptive immune responses (213, 214). Belatacept proved able to decrease the antigraft humoral immune response in intracerebral transplantation of mesencephalic pig xenografts into primates (215). *In vivo*, CTLA4Ig is able to dampen T cell-dependent immune responses and prolong long-term xeno- and allograft survival (216–218). Levisetti et al. reported that two out of five CTLA4Ig-treated monkeys showed prolonged graft survival, while the humoral responses were suppressed in all treated animals (219). Buerck et al. and his group generated a novel transgenic (tg) pig line expressing the CTLA-4Ig analogue LEA29Y and demonstrated that transplanted INSLEA29Y-tg porcine neonatal porcine islet-like clusters (NPICCs) displayed normal beta cell function and survived from rapid T lymphocyte-mediated rejection during 30-day observation period. However, the long-term effect regarding xenograft rejection still remained unknown (220). In another pig to baboon clinical trial, the blockade of CD28-B7 costimulation pathway using human CTLA4Ig has been shown unsuccessful to prevent xenograft rejection, making the role of CTLA4Ig controversial (221).

#### 3.5.2 Targeting of CD154:CD40 costimulatory signal with anti-CD40mAb

The interaction between CD154 on activated T cells and CD40 on APCs results in CD80/86 upregulation on APCs, enhancing another component of T cell co-stimulation (222). CD154 is also found on platelets, and not surprisingly drugs targeting CD154 are associated with an increased risk of thrombosis in primate (223). Thus, the focus of drug development has been changed to target CD40 and several anti-CD40mAbs are under development. Blockade of CD40/CD154 signaling by anti-CD40mAb was shown to prolong graft survival and suppress xenograft rejection (192, 224). Among these, a fully humanized anti-CD40mAb, iscalimab, appears to be a promising candidate in transplantation (225). Pre-clinical application using immunosuppressive anti-CD40mAb 2C10R4 combined with tacrolimus in pig-to-NHP islet xenotransplantation was effective in prolonging islet graft survival (164). In pig-to-mouse islet xenotransplantation, short-term administration of the anti-CD40mAb MR-1 and the anti-LFA-1mAb increased the survival of neonatal porcine islets (226). Interestingly, the short-term use of MR-1 alone prolonged porcine islet graft survival and promoted CD4+ Tregs recruitment into the graft and secondary lymphoid tissues (227). Consistently, lower numbers of CD4+ Tregs increased the risk of rejection in cardiac xenotransplantation in a pig-to-NHP model (228). For treatment, anti-CD40mAb was reported to be infused intravenously at a dose of 20–50 mg/kg on days –4, 0, 4, 7, 10 and 14 of transplantation, followed by weekly infusion for three months, and biweekly fusion thereafter (164). Alternatively, anti-CD40mAb could be used at a dose of 50 mg/kg on days –1, 0, 5, 9, and 14 (206).

### 3.6 Complement inhibition

As discussed above, complement activation is involved at every stage of xenograft rejection. Thus, a complementary approach is to administer agents that either deplete or inhibit complement activation. Many interventions have been introduced to prevent complement-mediated injuries during xenotransplantation (229, 230). Among these, cobra venom factor extends graft survival significantly in allotransplantation, albeit it only has a temporary effect (231). C1-esterase inhibitor has been reported to be active in NHPs and was recommended to replace cobra venom factor as complement inhibitor (232, 233). The compstatin analogue Cp40, a newly developed potent inhibitor of complement C3, inhibits leukocytes adhesion and neutrophils attachment to porcine endothelium (230). Moreover, Cp40 inhibits pECs and leukocytes activation. It reduces the levels of adhesion molecules such as E-selectin, ICAM-1, ICAM-2, and VCAM-1 on pECs, and of the integrin CD11b on neutrophils, paving the way for future therapeutic interventions targeting complement activities (230). Schmitz et al. reported that Cp40 could significantly prolong median allograft survival time in an NHP model. Normal kidney function was maintained at 50% in Cp40-treated primates after the last day of treatment (234). In another case report, Tibetan macaques receiving liver xenografts with immunosuppressors, including Cp40, did not exhibit severe coagulation disorders or immune rejection (235). Another complement inhibitor, the anti-C5 antibody Tesidolumab, has been recently reported to reduce early antibody-mediated rejection and prolong survival in renal xenotransplantation (236). Cp40 was used at a dose of 2 mg/kg three times daily, on day 2 prior to kidney transplantation and day 14 after kidney transplantation (234). Yet, the most appropriate dosages need to be determined more precisely. Tesidolumab was given at a dose of 30 mg/kg on the day of transplant, followed by weekly intravenous injection at 10 mg/kg for seven weeks, at which point anti-C5 was discontinued.

### 3.7 Genetic engineering strategies

Currently we are able to create new genetic modifications of the porcine genome (over 40 genetic variants to date), hoping to achieve better survival of the xenografts. The multiplex creation of a *GGTA1/CMAH/B4GalNT2* KO pig has shown the ability to reduce antibody mediated rejection in humans. There are also other extensive genome engineered pigs which have greater compatibility with the human immune system. For example, the generation of porcine endogenous retroviruses (PERVs) KO·3KO·9TG (hCD46, hCD55, hCD59, hTHBD, hTFPI, hCD39, hB2M, HLA-E and hCD47) pig enhances the pigs’ immunological compatibility and blood-coagulation compatibility with humans (64, 237–240). Recombinant expression of human complement regulatory molecules hCD59 and hDAF on porcine articular chondrocytes could also prevent humoral rejection in cartilage repair (241). By using CRISPR/Cas9 system, Sake et al. tried to abrogate MHC-I expression on xenografts to silence T-cell and NK cell-mediated cell lysis (64). Four genetic pigs died within the first days due to weakness, and the remaining two piglets developed acute fevers at an age of 3-4 weeks leading to sudden death (64). Xie et al. succeeded in alleviating antibody-mediated rejection using Gabarapl1 knockdowns in primary porcine aortic endothelial cells (PAECs) (238). While in another pre-clinical trial, the inhibition of COX-2 expression decreased PAECs death from 20% to 7% after 2 hours, making COX-2 inhibitors a candidate for therapeutic targeting to protect vascular endothelial cells in xenotransplantation (43). More is not always better, extensive genetic engineering can lead to congenital malformations/decrease animal viability (242, 243). What exact genetic modifications do we need in the organ-source pig should be fully considered in the future.

It is noteworthy that researchers are investigating alternative options. One of them is to grow complex tissues or organs using the body’s own regenerative capacity. Masano et al. tried to use a xenogeneic animal as an *in vivo* bioreactor to promote regeneration of a liver graft and successfully acquired fully regenerated small liver grafts under appropriate immunosuppressive therapy (244). This alternative option has fewer ethical concerns, but before it can be considered further, more research are needed to reduce complications and tested in larger animal models.

## 4 Organ-specific barriers and challenges

In recent years, a number of pig-to-NHP preclinical xenotransplantation studies have been performed with various organs. While HAR has been alleviated owing to gene-editing technologies, DXR and chronic rejection remain urgent issues to be solved. Another concern focuses on PERVs transmission, which is a major hurdle to the clinical use of pig cells, tissues, and organs for treatment of organ failure in humans. However, it is still uncertain whether PERVs is pathogenic to humans, or if it could recombine with hERVs to form new viruses. Genetic engineering techniques such as CRIPSR/Cas9 genome editing system could prevent their activation or delete them from the pig cells (245, 246). Further, the ethical issues around xenotransplantation have not been sufficiently discussed. Strict medical and ethical guidelines and regulations are needed before clinical applications can be tried on selected patients. Beyond the common ethical and technical issues shared by the different areas of xenotransplantation, there are also organ-specific barriers that are briefly addressed below.

### 4.1 Islet xenotransplantation

Islet xenotransplantation is a promising alternative approach to Type 1 Diabetes (T1D) treatment and has achieved long-term normoglycemia in porcine-to-primate studies (247–249). Based on preliminary studies in NHPs, the first case of clinical islet xenotransplantation to human can be traced back to 1994 (250). Currently, clinical trials using islet xenotransplantation are developing more rapidly than those in other xenotransplantation areas. In 2014, a clinical trial using islet xenotransplantation under regulatory framework was registered at ClinicalTrial.gov (251). This was followed by Phase I/IIa and IIb clinical trials using encapsulated neonatal porcine islets in xenotransplantation performed in Argentina (252), which resulted in a mean transplant estimated function of approximately 0.5, with transplants maintained for more than two years, and a significant reduction in the number of unaware hypoglycemia episodes. Surely, there are still many problems to be solved for islet xenotransplantation before reaching their clinical use, including physiological function consistency, immune rejection, islet loss, and prevention of PERVs infection (253). Immune rejections included HAR, mediated by Gal and non-Gal antigens (254), instant blood-mediated inflammatory reaction (IBMIR) that may have provoked 60–80% of islet loss (255), and CD4^+^ T cell-mediated cellular rejection that plays a major role in islet destruction (256). Strategies to alleviate rejection mainly include islet encapsulation and gene editing technology. Recent clinical trials mainly focused on encapsulating the neonatal porcine islets in different high molecular compounds (257). While cell encapsulation technology can potentially shield the islets from the host’s immune rejection at initial stage, long-term therapeutic efficacy is still a challenge (258). Another hot spot of research is to attempt different transplantation sites, including the portal vein, subrenal capsule, subcutaneously, the muscle, spleen, gastric submucosal space and peritoneum, depending on the islet volume and its naked or encapsulated status, in order to reduce islet loss (259, 260). There is still no optimal site for transplantation, but the peritoneal cavity is favoured in clinical trials.

### 4.2 Liver xenotransplantation

Liver xenotransplantation from chimpanzee to human was first held in the 1960s (261). More trials were carried out in the 1990s (262), which remained ultimately unsuccessful. The transplanted patients either died from sepsis due excessive immunosuppression, or from hepatic failure with clear rejection. These failures terminated the attempts of liver xenotransplantation in clinical application. Until today, the research has mainly focused on pig liver xenotransplantation to NHPs. Shah et al. (263) have recorded, so far, the longest survival time after xenotransplantation of a pig livers to NHPs, which was of 25 days, using with an *α1,3-galactosyltransferase* knockout miniature swine as a donor. Two major problems must be overcome in liver xenotransplantation: lethal thrombocytopenia and antibody-mediated rejection (AMR) targeting antigens such as *α1,3GT*, N- glycolylneuraminic acid and *β4GALNT2* (235). Moreover, hepatic cold-induced injuries are also a serious concern (264). AMR has now been reduced by using gene editing technology including CRISPR/Cas9, TALEN, and other genome editing and transgenic methods (254). To solve hepatic cold‐induced injuries, Li et al. succeeded to increase porcine hepatocyte viability by optimizing spheroid cold storage conditions under four different cold storage solutions (265). Despite these efforts, the graft survival is limited by either the development of a thrombotic microangiopathy and/or consumptive coagulopathy (266, 267). Cross-species thromboregulation becomes more complicated in case of liver xenotransplantation because the liver produces most coagulation factors. Current preclinical studies are dedicated to elucidating the immunobiology behind platelet activation, aggregation, and phagocytosis, especially during interactions between platelets and liver sinusoidal endothelial cells, hepatocytes, and Kupffer cells (28, 268). We believe that if the severe and immediate thrombocytopenia could be prevented, pig liver xenotransplantation could be used as a bridge towards allotransplantation.

### 4.3 Cardiac xenotransplantation

The first attempt of pig-to-NHP cardiac xenotransplantation (CXTx) started in the mid-1980s (269). Currently, it is the standard model to conduct preclinical xenotransplantations. A major breakthrough came with the introduction of the genetic deletion of the *α1,3GT* gene in 2003, which reduces HAR to a large extent. The longest survival of heterotopic heart xenograft, reaching up to 945 days, has been achieve in baboons, using cardiac xenografts from *GTKO.hCD46.hTBM* pigs, with ATG and anti-CD20 antibody treatments, followed by maintenance with MMF and high-dose anti-CD40 immunosuppressive regimen (193). The first clinical trial of pig to human CXTx was carried out with genetically modified pig heart transplanted into a 57-year-old man in the USA in 2022, and the patient survived for two months without signs of rejection, while the cause of death is unknown (270). Despite many breakthroughs on different aspects of xenotransplantation in recent years, there are still barriers to be overcome before large scale clinical CXTx can be conducted, including immunological barriers, perioperative cardiac xenograft dysfunction (PCXD), detrimental xenograft growth, and PERV infection. PCXD is unique to orthotopic CXTx and has not been observed in heterotopic CXTx. It can cause xenograft failure within the first 48 hours (194). The exact mechanism of PCXD is unclear but may stem in incompatibilities between porcine and primate plasma, the latter carrying non-Gal antibodies (271). Cold non-ischemic continuous perfusion of the donor’s heart with STEEN solution (a buffered extracellular solution) appears to be an effective way to alleviate PCXD (233, 272). The detrimental xenograft overgrowth occurring after CXTx leads to diastolic dysfunction and congestive liver damage. The overgrowth could be inhibited by lowering baboons’ blood pressure to match that in pigs’ heart, by reducing the use of cortisone early, or by using temsirolimus, as in a particular study (233). This latter strategy has not tested by other researchers. The relevant ethical issues around CXTx have not been completely defined yet. Strict medical and ethical guidelines and regulations are still needed before proceeding towards clinical application to selected patients.

### 4.4 Kidney xenotransplantation

Kidney xenotransplantation has a long and largely unsuccessful history. The first clinical trial was carried out in 1905, when slices of rabbit kidneys were inserted into a child, who died 16 days later due to pulmonary congestion. Reemtsma et al. transplanted pairs of chimpanzee kidneys into six patients in 1964, with the longest survival reaching nine months (273). Later attempts using monkeys and baboons as source of kidneys were even less successful. In majority, the deaths occurring in these clinical studies were related to either rejections or infections. These disappointing results terminated the clinical application of kidney xenotransplantation. However, researchers have re-explored this possibility later, due to shortage of available kidneys, and pig-to-NHP xenotransplantation has now become a standard experimental model. Similar to CXTx, rapid progress has been made since 2005, with the availability of genetically engineered pigs (274). In 2015, two groups reported the survival of life-supporting genetically engineered pig kidneys for > 4 months, maintained by a treatment involving new immunosuppressive agents blocking T cell co-stimulation (201). Based on this progress, a new clinical trial with two GTKO porcine kidneys transplanted to a brain-dead patient on a ventilator has been carried out in 2021. The kidneys were connected outside of the body to blood vessels on the patient’s legs and monitored over a period of 72 hours (275). No HAR and no transmission of porcine retroviruses were detected, and the kidneys produced variable amounts of urine, but creatinine clearance was not recovered. Although this attempt surely brought substantial information and improvement for kidney xenotransplantation, there is still a long way to make kidney xenotransplantation possible in humans. In this case, the host was brain dead and artificially maintained, and therefore cannot been considered a living body. This trial is close to a clinical trial, but it still cannot be considered as such. We would consider this attempt as a bridge between xenotransplantation trials in animals and clinical trials in humans. Organ-specific problems linked to kidney xenotransplantation include hypovolemia syndrome, erythropoietin function-associated anaemia, and rapid growth of the pig kidneys after transplantation (199). The primate organisms are not aware of the fluid loss occurring during hypovolemia syndrome, which may result from a dysfunction of the renin-angiotensinogen system. This could be avoided by conserving the native kidneys *in situ* (276). It is difficult to assess whether the pig erythropoietin functions adequately in primates, but pigs genetically engineered to produce human erythropoietin may solve this issue (277). Kidneys transplanted from a strain of pig grow early and rapidly in primates. This phenomenon may result from an innate factor, and could also be solved by knocking out the gene encoding the growth hormone receptors of the pigs (278). Finally, the inclusion criteria for the selection of patient candidate to kidney xenotransplantation is more difficult than in other areas, as dialysis represents a therapeutic option for these patients. Therefore, until proven safer and more efficient, kidney xenotransplantation cannot be considered.

## 5 Conclusion and perspectives

The field of xenotransplantation has been progressing rapidly with many breakthrough achievements in recent years. However, there are still several problems ahead of its use in clinical practice: (i) although porcine-to-human xenotransplantation of kidneys and hearts have been carried out, it is not known when xenotransplantation of liver, small intestine, and even pancreas will become possible. (ii) NHPs are phylogenetically close to humans and share many physiological, anatomical, immunological, and neurological similarities, making them excellent experimental models for research (279). However, there are still differences between species, and it is not clear to which extent studies in NHPs can fully predict xenorejection and the clinical outcome in humans. (iii) Until now, the survival of transplanted organs on the long term largely depends on high doses of different immunosuppressants, which would expose the recipients to high infection risks and other side effects. Although more and more genetically engineered pigs are created by gene-editing technologies, the question remains as to whether it will become possible to achieve long-time survival without immunosuppressants, by reducing the immunogenicity of the transgenic donors. Ethical aspects of xenotransplantation have been discussed for many years (280), with particular considerations on issues related to the risks for patient with xenograft do develop and propagate porcine infections (281). (iv) While CRISPR/Cas9 genome editing can remove the PERV gene from pig, the risk of xenozoonosis with other roseoloviruses remains (282). For example, porcine cytomegalovirus was the cause of a significant reduction of the survival time of the transplanted pig organs (283). However, such concerns can also be appropriately handled with modern drug therapies, selective breeding, and genetic modification.

As allotransplantation is restricted due to cell and organ shortage, xenograft provides an alternative source of tissues, and xenotransplantation may represent the next revolution in medicine. More patients with end-organ failure would undoubtedly benefit from breaking the immunological barriers to xenotransplantation in near future.

## Author contributions

YW, CC and SD conceived the idea. QZho and TL wrote the manuscript. KW and QZha prepared the figures. CC, KW, ZG, CC and YW revised the manuscript. All authors contributed to the article and approved the submitted version.

## Funding

This study was supported by an NSFC grant (No. 81802504), the International Innovation Cooperation Project of Sichuan Science and Technology Bureau (2022YTH0005 and 2019YFS0439), the Science and Technology Innovation Project of Chengdu, China (No. 2021-YF05-00225-SN), a Sichuan Medical Association grant (No. Q19037) to YW, the Science and Technology Bureau of Sichuan Province for outstanding young talent (No. 2020JDJQ0067) to QZho, and the Pelotonia Postdoc Fellowship and an OSU Department of Radiation-Oncology Basic Research seed grant to CC.

## Conflict of interest

The authors declare that the research was conducted in the absence of any commercial or financial relationships that could be construed as a potential conflict of interest.

## Publisher’s note

All claims expressed in this article are solely those of the authors and do not necessarily represent those of their affiliated organizations, or those of the publisher, the editors and the reviewers. Any product that may be evaluated in this article, or claim that may be made by its manufacturer, is not guaranteed or endorsed by the publisher.
